# HIV-1 Transgenic Rats Display Alterations in Immunophenotype and Cellular Responses Associated with Aging

**DOI:** 10.1371/journal.pone.0105256

**Published:** 2014-08-15

**Authors:** Susan J. Abbondanzo, Sulie L. Chang

**Affiliations:** Institute of NeuroImmune Pharmacology and Department of Biological Sciences, Seton Hall University, South Orange, New Jersey, United States of America; University of Nebraska Medical Center, United States of America

## Abstract

Advances in anti-retroviral therapy over the last two decades have allowed life expectancy in patients infected with the human immunodeficiency virus to approach that of the general population. The process of aging in mammalian species, including rats, results in immune response changes, alterations in immunological phenotypes, and ultimately increased susceptibility to many infectious diseases. In order to investigate the immunological pathologies associated with chronic HIV-1 disease, particularly in aging individuals, the HIV-1 transgenic (HIV-1Tg) rat model was utilized. HIV-1Tg rats were challenged with lipopolysaccharide (LPS) to determine immunological alterations during the aging process. LPS is known to cause an imbalance in cytokine and chemokine release, and provides a method to identify changes in immune responses to bacterial infection in an HIV animal model. An immune profile and accompanying cellular consequences as well as changes in inflammatory cytokine and chemokine release related to age and genotype were assessed in HIV-1Tg rats. The percentage of T cells decreased with age, particularly T cytotoxic cells, whereas T helper cells increased with age. Neutrophils and monocytes increased in HIV-1Tg rats during maturation compared to age-matched F344 control rats. Aging HIV-1Tg rats displayed a significant increase in the pro-inflammatory cytokines, IL-6 and TNF-α, along with an increase in the chemokine, KC/GRO, in comparison to age-matched controls. Our data indicate that immunophenotype and immune responses can change during aging in HIV-positive individuals. This information could be important in determining the most beneficial age-dependent therapeutic treatment for HIV patients.

## Introduction

Life expectancy of people infected with the human immunodeficiency virus (HIV) is increasing and approximates that of the general population. The current median life expectancy for an HIV patient receiving effective therapy exceeds 70 yrs of age [1]. In the United States, estimates indicate that, by 2015, most of the patients diagnosed with HIV will be 50 yrs of age and older [2]–[4]. A recent publication from the United Kingdom reported that the percentage of HIV-positive individuals who are over 50 yrs of age has risen from 12% in 2002 to 22% in 2011 due to an increase in the length of survival and a surge in the incidence of newly identified older HIV patients [5]. Furthermore, a majority of the older patients were aware of their HIV status for at least a decade, and were being treated with anti-retroviral therapy since testing positive. However, as the population of HIV-positive patients ages, they often develop other ailments, including cardiovascular disease, cancer, osteoporosis, and liver and renal disease, as well as the neurocognitive deficiencies more commonly associated with aging and chronic inflammation than with HIV infection [6]–[12].

The process of aging in many mammalian species, including rats, results in changes in immune responses, alterations in immunological phenotypes, and ultimately increased susceptibility to infectious diseases, cancer, and autoimmune disorders [13]–[16]. Unraveling the mechanisms underlying the immunophenotypic changes associated with human HIV infection is now possible utilizing a small animal model, the HIV-1 transgenic (HIV-1Tg) rat [17]. This animal model possesses human viral genes, including the HIV-1 pro-virus, but with the deletion of the *gag* and *pol* replication genes. The HIV-1Tg rat exhibits similar clinical manifestations as HIV-positive humans, including wasting, skin lesions, cataracts, neurological and respiratory impairment, and changes in the immune system [17]–[20], suggesting that the HIV-1Tg rat model is useful for studying HIV-1-infected patients on highly active anti-retroviral therapy (HAART) [21]. In the last decade, many reports have demonstrated that the HIV-1Tg rat is also a valuable model for studying neuroAIDS [20], [22], [23]. The HIV-1Tg rat is, therefore, an ideal small animal model for studying the immunological alterations and pathology associated with chronic HIV-1 infection during aging.

Viral gene expression has been detected in the lymph nodes, spleen, thymus, and blood of the HIV-1Tg rat, indicating that this animal model exhibits alterations in immune cell responses [17], [18]. HIV-specific transcripts (7-, 4-, and 2-kb) are present in the spleen, kidney, and thymus, and highest in the axillary lymph nodes. Further analysis revealed that the HIV protein, gp120, is present in macrophages as well as B and T cells derived from splenic lysates. In addition, it has been reported that the viral proteins, Tat, gp120, Nef, and Vif, in the spleen of 2–3 mo old HIV-1Tg rats is higher than in 10–11 mo old animals [21]. Although the HIV-1Tg rat spleen is normal in size, it does exhibit a loss of cells in the periarterial lymphatic sheath (PALS), larger marginal zones, follicular hyperplasia, and apoptosis of endothelial cells. However, when immunized with keyhole limpet hemocyanin (KLH) to analyze immune cell function, there is no significant difference in anti-KLH-specific titers and a reduced induration in the delayed type hypersensitivity (DTH) response, suggesting that the HIV-1Tg rats have abnormal Th1 (T helper) responses, but normal Th2 responses [17], [18].

In older HIV-1Tg rats (12–15 mo old), the CD4^+^ T cell population displays altered CD28 function, reduced anti-apoptotic Bcl-xL expression, reduced IL-2, and increased apoptosis [24]. Mature HIV-1Tg rats also have decreased CD4^+^ and CD8^+^ effector and memory cells and an increased number of naïve cells [17], [18]. These findings suggest that aging may influence immune function of T cell populations in the HIV-1Tg rat.

In order to explore the effect of aging on immune cell responses, determine the phenotypic changes associated with genotype, and establish immunophenotypic profiles in this small animal model of HIV, a polychromatic flow cytometry approach was employed in this study. Wild-type F344 and HIV-1Tg rats were grouped by age, from 2 mo, 5–6 mo, to 18–20 mo. Whole blood and spleen samples were analyzed for changes in immune cell populations, including B cells, T cells, T helper cells, T cytotoxic cells, neutrophils, and monocyte subtypes, classical and non-classical, as defined in recent publications [25], [26].

It has been reported that HIV-1Tg rats also display altered pro-inflammatory and anti-inflammatory cytokine and chemokine expression [27]. This led us to investigate whether the immune cell profile and responses are compromised during aging in the HIV-1Tg rat. Using an endotoxin tolerance (ET) model, animals at different ages were rendered tolerant by exposure to two low doses of lipopolysaccharide (LPS), then challenged with a high dose of LPS as previously described [27], [28]. Blood, spleen, and lymph nodes of the HIV-1Tg rats and age-matched F344 control rats were analyzed for changes in immunophenotype related to age and genotype. In addition, age-related changes in immune function were determined by examining cytokine and chemokine production in the LPS treated animals.

The results of this study will provide evidence of the effects of aging on the immune cell profile and function in the HIV-positive population compared to non-infected individuals. This information will be helpful in determining the best treatment for HIV-infected individuals based on multiple factors, including immune cell profiles, cellular responses, and the patient's age.

## Materials and Methods

### Animals

All animals were purchased from Harlan Co. (Indianapolis, IN) at approximately 4–8 wks of age, and maintained in ventilated cages up to 20 mo of age. Male wild-type (F344) and HIV-1 transgenic (HIV-1Tg/F344) rats were grouped according to age. All animal experiments were carried out in Seton Hall University's Animal Care Facility. Animal care and experiments were performed in accordance with the Animal Welfare Act and Public Health Service Policy. Approval was obtained from the Institutional Animal Care and Use Committee at Seton Hall University prior to the start of experiments. Euthanasia, when necessary, was performed using carbon dioxide asphyxiation and cervical dislocation.

### Flow cytometry

Whole blood was collected at 2, 5–6, 12, and 18–20 mo of age from the rat tail vein into Becton Dickinson (BD) lithium heparin tubes (BD 365965). Twenty microliters of blood was blocked for non-specific staining with 0.25 µg anti-rat CD32 (BD 550271) for 5 min. Fluorescently labeled antibodies were added according to the manufacturer's protocol. The cells were treated with antibodies and labeled as follows: B cells (CD45RA, BD 561624), T cells (CD3, BD 557354), T helper cells (CD4, BD 554839), T cytotoxic cells (CD8a, BD 558824), neutrophils (RP-1, BD 550002), monocyte subtypes (CD43-AF647, Biolegend 202810; CD172, BD 552298; CD11b, BD 562105), and isotype-matched gating controls. The cells were treated for 30 min at 4°C with fluorescently labeled antibodies, and then centrifuged for 5 min at 300*xg*. The supernatant was removed and the blood was treated with 1X cell lysing solution (BD 349202) for 10 min at room temperature (RT). The cells were centrifuged for 5 min at 300*xg*, then washed with FACS buffer (PBS, 0.1% BSA, 25 mM HEPES). All samples were re-suspended in FACS buffer, prepared in duplicate, and 30,000 events were acquired on the BD Fortessa. Immune cell populations were gated as follows: B cells (CD45RA^+^/CD3^−^), T cells (CD3^+^/CD45RA^−^), T helper cells (CD3^+^CD4^+^), T cytotoxic cells (CD3^+^CD8^+^), neutrophils (RP-1^+^), and classical (mononuclear/CD172^+^/CD43^+^) and non-classical (mononuclear/CD172^+^/CD43^++^) monocytes, as described previously [25].

Spleen samples were collected from 2, 5–6, and 18–20 mo old animals at the time of sacrifice. Spleen cells were isolated by pulverizing the spleen tissue through a 100 µm nylon mesh (Fisher, 22363549), washing the cells in FACS buffer, then treating them with red blood cell lysing buffer (Gibco #A10492-01) for 5 min at RT. The cells were washed with FACS buffer, centrifuged, and counted. The spleen cells (10^6^ cells/sample) were stained with fluorescently labeled antibodies as described for whole blood.

### Lipopolysaccharide (LPS) administration

Male HIV-1Tg and F344 rats were grouped by age and genotype (N = 5/group): 2, 5–6, and 18–20 mo of age. Treatment with LPS to induce ET in each group was completed as previously established in our laboratory [27], [28]. Briefly, the animals were administered an intraperitoneal (i.p.) non-pyrogenic 250 µg/kg dose of LPS (from *E. Coli* 055:B5, Sigma, L2880) on Day 1 at 0 h and 10 h, and a challenge injection of 5 mg/kg LPS at 24 h. Animals treated with LPS are termed “LPS treated”. Age- and genotype-matched animals were injected with 0.9% saline (designated as ‘control’) on the same time schedule as the LPS treated rats.

### Cytokine and chemokine analysis

To measure cytokine levels in the LPS-treated animals, the rats were sacrificed 2 h after the final LPS injection (at 26 h). The spleen and lymph nodes (axillary and mesenteric) were isolated and stored at −80°C. The tissue was homogenized in lysis buffer containing 5 mM Tris-HCl (Boston BioProducts, BM-320), 2 mM EDTA (Boston BioProducts, BM-150), 1% Triton X-100 (Sigma, T6878), and 1 vial of protease inhibitor cocktail (Sigma P2714) at a ratio of 1∶10 (tissue:buffer). The samples were homogenized with a Polytron PT2100 for 30 s on ice, centrifuged at 6,800 *rcf* for 5 min at 4°C, and the supernatants were collected. Total protein concentrations were measured using a micro BCA Protein Assay Kit (Pierce #23235). Blood was collected into serum separation tubes (BD, 365967), spun down, and the serum was separated. Serum (30 µl) was diluted 1∶4 with Diluent 42 (MesoScale Discovery).

The levels of ten cytokines and chemokines [interferon (IFN)-γ, IL-1β, IL-2, IL-4, IL-5, IL-6, KC/GRO, IL-10, IL-13, tumor necrosis factor-α (TNF-α)] were determined from 100 µg of spleen or diluted serum using the rat pro-inflammatory V-plex kit (panel 1, MesoScale Discovery), according to the manufacturer's instructions. Due to limitations in the amount of lymph node tissue, only IL-6 and TNF-α levels were examined in 50 µg of lymph node total protein. All samples were run in duplicate, and the mean and standard deviation was calculated.

### Western blot analysis

The tissues were homogenized and total protein concentrations determined as described above. Protein (40 µg) was added to 2X LDS sample buffer (Invitrogen, NP0007, 4X diluted 1∶1 with water) with 5% reducing agent (Invitrogen, NP0004), and heated for 7 min at 95°C. Proteins were resolved on a 4–12% Bis-Tris gel (Novex #NP0322) and transferred onto a nitrocellulose membrane (Novex, 1B3011002) using the Ibot system. Membranes were blocked for 1 h at RT with TBS (Tris-buffered saline, ph 7.4, Boston BioProducts, BM-300) containing 0.05% Tween 20 (Fisher, BP337-500) and 1% bovine serum albumin (BSA, Gemini, 700-100P). Membranes were incubated with primary TNF-α (1∶1000, Abcam, ab66579) or IL-6 (1∶1000, Abcam, ab6672) antibody, with β-actin (1∶1000, cell signaling #8H10D10) as a loading control, followed by secondary antibodies for visualization (1∶15,000, goat anti-mouse IgG, Licor 827-08364; donkey anti-rabbit IgG, Licor 926-68073) diluted in blocking buffer (Licor, 927-40000). Proteins were analyzed on the Odyssey infrared imaging system for detection and quantification. The integrated intensity signal of β-actin was used to normalize the signal of TNF-α and IL-6. The percentage change in normalized protein was calculated by comparison to the F344 age-matched control.

### Statistical analysis

Flow cytometry analysis was performed using DIVA 6.1.3, FlowJo V10, and Graph Pad Prism 5 software. All data are presented as the mean ± SD. Statistical differences among the groups were assessed by a one-way ANOVA, and post hoc multiple comparisons were performed using the Bonferroni multiple comparisons test. The significance level was set at p<0.05.

## Results

### Immunophenotypic analysis of untreated peripheral blood cells in aging rats

**Figure 1:**
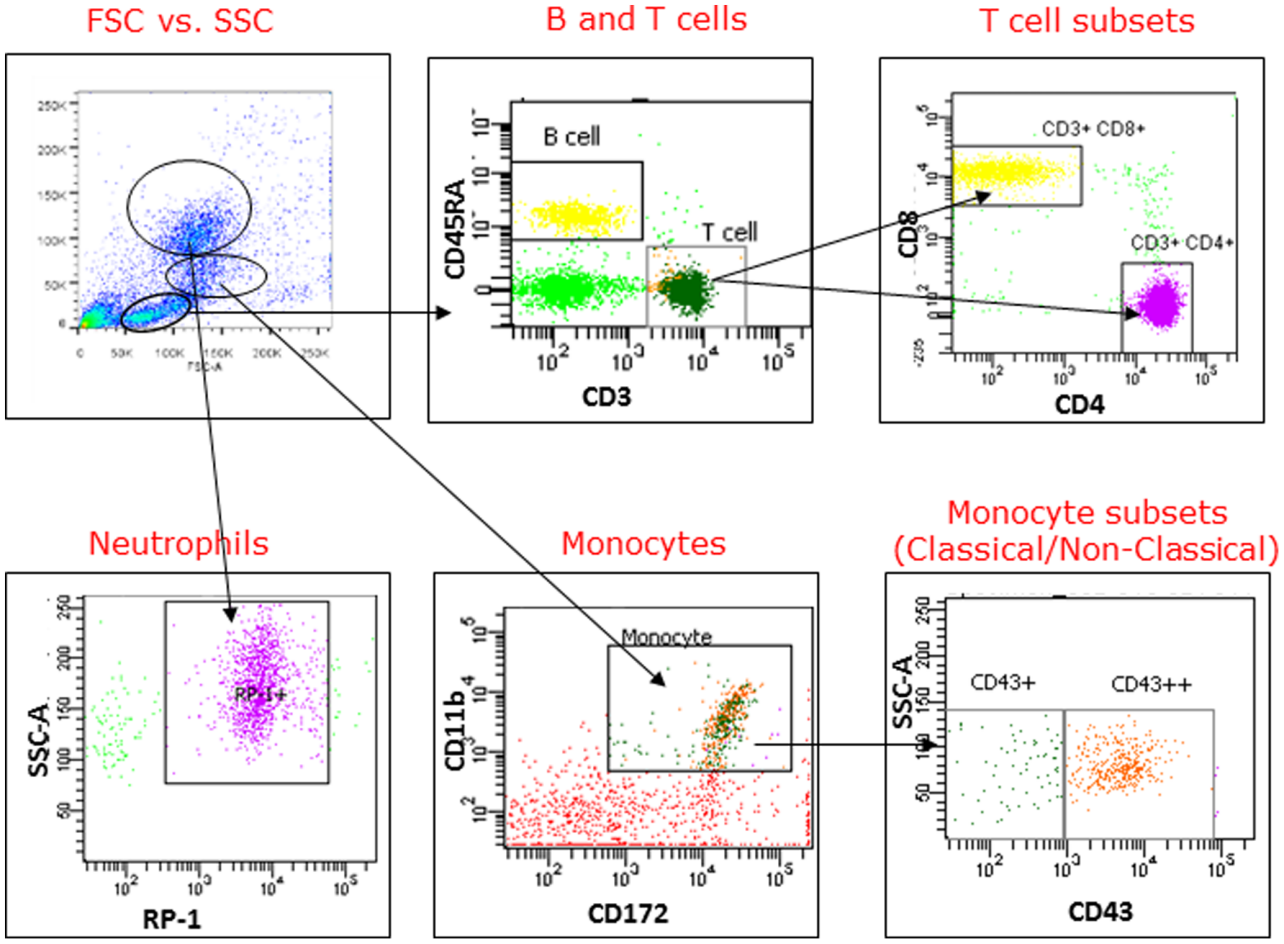
Flow cytometry gating strategy for HIV-1Tg and F344 rat blood. Mononuclear, lymphocytes, and granulocytes were gated on FSC versus SSC. Cell surface antibodies were used to identify B and T cells, T cytotoxic cells, T helper cells, neutrophils, monocytes, and monocyte subtypes (classical and non-classical).

Immune cell populations in whole blood were gated as illustrated in Fig. 1.

**Figure 2:**
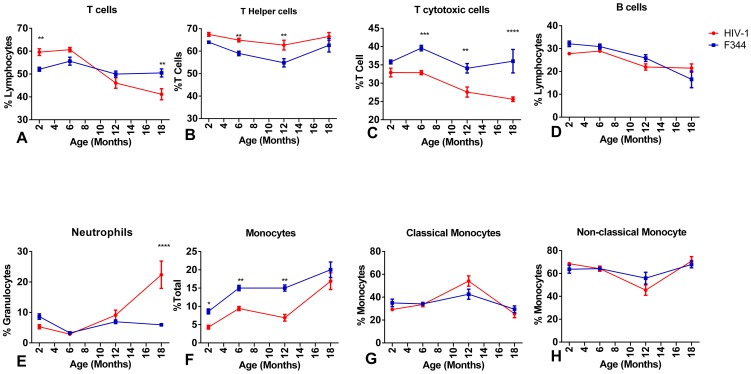
Analysis of cell populations in whole blood from HIV-1Tg and F344 rats. Flow cytometry analysis of untreated HIV-1Tg immune cell populations (red line) and untreated F344 age-matched rats (blue line). (A) T cells (CD3+); (B) T helper cells (CD3+/CD4+); (C) T cytotoxic cells (CD3+/CD8+); (D) B cells (CD45RA+); (E) neutrophils; (F) monocytes; (G) classical monocytes (CD43+); (H) non-classical monocytes (CD 43++). *p<0.05, **p<0.01, ***p<0.001, ****p<0.0001.

CD3^+^ T cell lymphocytes (Fig. 2A) were significantly increased in 2 mo old HIV-1Tg rats compared to age-matched F344 animals. However, T cell percentages decreased in both groups during aging, but to a greater extent in 18 mo old HIV-1Tg rats. The percentage of T helper cells (CD3^+^/CD4^+^) from the HIV-1Tg rats was higher throughout the aging process, with a significant increase in 5–6 and 12 mo old animals (Fig. 2B). There was a significantly lower percentage of T cytotoxic cells (CD3^+^/CD8^+^) in the 6, 12, and 18 mo old HIV-1Tg rats compared to the age-matched control rats (Fig. 2C).

Flow cytometry analysis revealed that the percentage of B cells in both the HIV-1Tg and F344 rats was higher in the 2 mo old animals and decreased with aging (Fig. 2D).

The percentage of neutrophils was low (<10%) in both the HIV-1Tg and F344 rats at 2, 5–6, and 12 mo of age. However, the percentage of neutrophils increased significantly in the 18–20 mo old HIV-1Tg rats compared to the age-matched controls (Fig. 2E).

There were a significantly lower percentage of monocytes (Fig. 2F) in the 2, 5–6, and 12 mo old HIV-1Tg rats compared to the F344 age-matched control animals, while the percentage of monocytes in the HIV-1 rats were similar at 18 mo. The percentage of classical monocytes in the HIV-1Tg rats increased at 12 mo of age compared to the control animals (Fig. 2G), whereas there was a slight, but not significant, decrease in the percentage of non-classical monocytes in the HIV-1Tg rats at 12 mo of age (Fig. 2H).

### Immunophenotypic analysis of peripheral blood cells in LPS-treated aging rats

**Figure 3:**
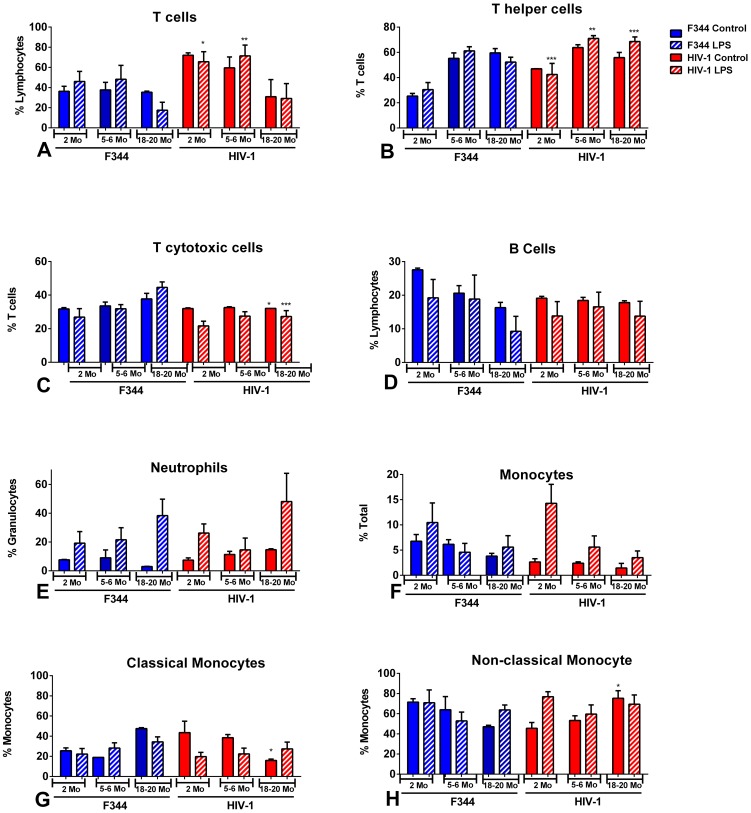
Analysis of cell populations in whole blood from aging HIV-1Tg and F344 rats, with and without LPS treatment. Flow cytometry analysis of HIV-1Tg cell populations (red bars) and F344 age-matched control rats (blue bars). Solid bars indicate control samples and bars with striped lines indicate samples that have been treated with LPS. (A) T cells; (B) T helper cells; (C) T cytotoxic cells; (D) B cells; (E) neutrophils; (F) monocytes; (G) classical monocytes; (H) non-classical monocytes. *p<0.05, **p<0.01, ***p<0.001, ****p<0.0001.

Flow cytometry analysis of whole blood showed that the percentage of CD3^+^ T cell lymphocytes was significantly higher in both control and LPS-treated 2 and 5–6 mo old HIV-1Tg rats compared to age-matched F344 rats (Fig. 3A), but displayed similar levels with 18 mo old HIV-1Tg and F344 rats. The percentage of T helper cells (CD3^+^/CD4^+^) in the LPS-treated HIV-1Tg rats was significantly higher throughout the aging process compared to the LPS-treated F344 rats (Fig. 3B). Conversely, the percentage of T cytotoxic cells (CD3^+^/CD8^+^) was significantly lower in the LPS-treated 5–6 and 18–20 mo old HIV-1Tg rats compared to the LPS-treated F344 animals (Fig. 3C).

The percentage of B cells was not significantly different between the age-matched HIV-1Tg and F344 rats or within the LPS treated groups (Fig.3D).

The percentage of neutrophils remained low in the blood of the control HIV-1Tg and F344 rats, but increased in response to LPS in both the HIV-1Tg and F344 rats in all age groups (Fig. 3E).

There was no significant difference in the percentage of monocytes in the control and LPS-treated HIV-1Tg rats compared to the age- and treatment-matched F344 groups. However, the percentage of classical monocytes was significantly lower in the 18–20 mo old control HIV-1Tg rats compared to the control age-matched F344 animals (Fig. 3G). Conversely, the percentage of non-classical monocytes was significantly increased in the 18–20 mo old control HIV-1Tg rats compared to the control age-matched F344 animals. (Fig. 3H).

### Immunophenotypic analysis of immune cells from the spleens of LPS-treated aging rats

**Figure 4:**
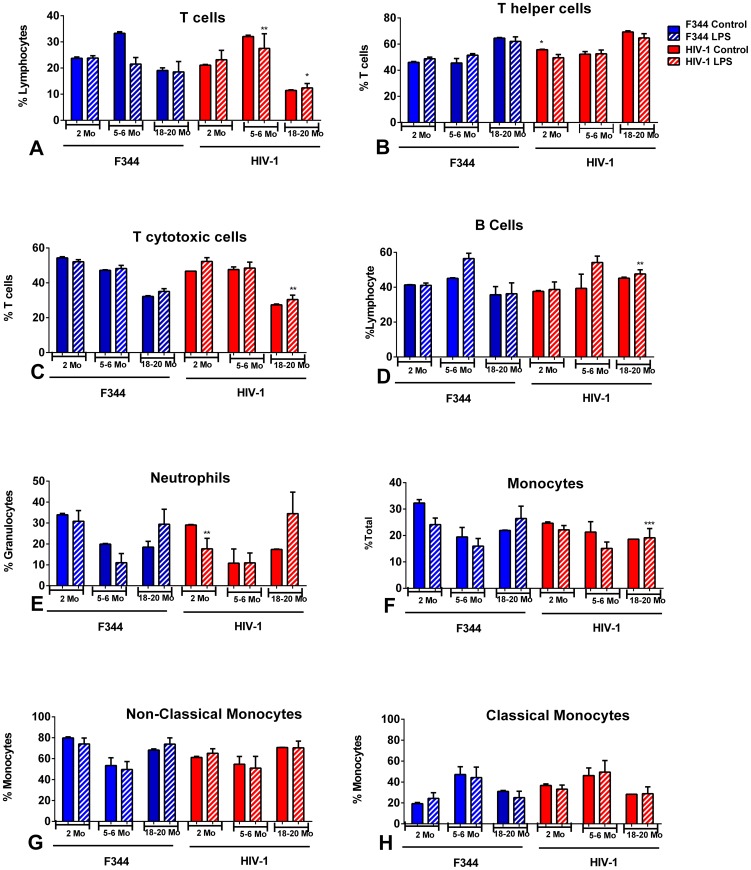
Immune cells in the spleens of aging HIV-1Tg and F344 rats, with and without LPS treatment. Flow cytometry analysis of immune cells from the spleens of HIV-1Tg rats (red bars) and F344 age-matched control rats (blue bars). Solid bars indicate control samples and bars with striped lines indicate samples that have been treated with LPS. (A) T cells; (B) T helper cells; (C) T cytotoxic cells; (D) B cells; (E) neutrophils; (F) monocytes; (G) non-classical monocytes; (H) classical monocytes. *p<0.05, **p<0.01, ***p<0.001, ****p<0.0001.

The distribution of immune cells in the spleens of the HIV-1Tg rats, with and without LPS treatment, was compared to age-matched F344 rats using flow cytometry analysis. The percentage of CD3^+^ T cell lymphocytes was significantly increased in 5–6 mo old LPS-treated HIV-1Tg rats and significantly decreased in the 18–20 mo old HIV-1Tg rats compared to the LPS-treated F344 animals (Fig. 4A). The percentage of T helper cells (CD3^+^/CD4^+^) was significantly higher in the 2 mo old control HIV-1Tg rats compared to the control age-matched F344 animals (Fig. 4B). There was a significantly lower percentage of T cytotoxic cells (CD3^+^/CD8^+^) in the 18–20 mo old LPS-treated HIV-1Tg rats compared to age matched LPS-treated F344 rats (Fig. 4C).

The percentage of B cells was significantly higher in the 18–20 mo old LPS-treated HIV-1Tg rats compared to the age-matched LPS-treated F344 rats (Fig. 4D).

The percentage of neutrophils in the spleen was significantly decreased in the 2 mo old LPS-treated HIV-1Tg rats compared to the age-matched LPS-treated F344 control animals (Fig. 4E).

There was a significantly lower percentage of monocytes in the 18–20 mo old LPS-treated HIV-1Tg rats compared to the LPS-treated age-matched F344 rats (Fig. 4F). However, the percentages of classical monocytes (Fig.4G) and non-classical monocytes (Fig 4H) were not significantly different when comparing age- and treatment-matched HIV-1Tg and F344 rats.

### Cytokines and chemokines in the serum, spleen, and lymph nodes of LPS-treated aging rats

**Figure 5:**
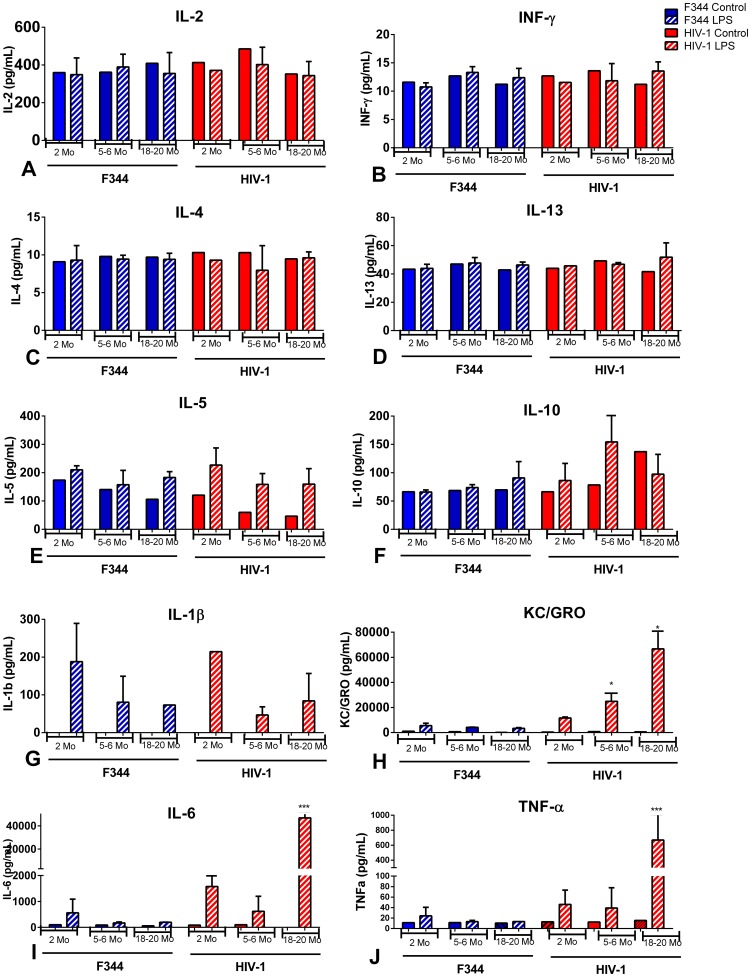
Cytokines and chemokines in the serum of aging HIV-1Tg and F344 rats, with and without LPS treatment. Cytokine and chemokine levels in serum from HIV-1Tg rats (red bars) and F344 age-matched control rats (blue bars). Solid bars indicate control samples and bars with striped lines indicate samples treated with LPS. (A) IL-2; (B) IFN-γ; (C) IL-4; (D) IL-13; (E) IL-5; (F) IL-10; (G) IL-1β; (H) KC/GRO; (I) IL-6; (J) TNF-α. *p<0.05, **p<0.01, ***p<0.001, ****p<0.0001.

The protein levels of IFN-γ, IL-1β, IL-2, IL-4, IL-5, IL-6, KC/GRO, IL-10, IL-13, and TNF-α in the serum, spleen, and lymph nodes of 2, 5–6, and 18–20 mo old HIV-1Tg and F344 age-matched rats treated with LPS or saline (control) were determined using an electrochemiluminescent assay. There was no difference in the levels of IL-2, INF-γ, IL-4, and IL-13 in the serum of the LPS-treated HIV-1Tg and F344 rats compared to the control animals (Fig. 5A-D). Although not significant, there was a slight decrease in the IL-5 levels associated with increased age in both the control F344 and HIV-1Tg rats; however, IL-5 was elevated in all the LPS-treated groups (Fig. 5E). There was no difference in IL-10 levels in any age group in either the control or LPS-treated F344 rats, and no difference in IL-10 in the 2 mo old control and LPS-treated HIV-1Tg rats. However, there was an increase, although not significant, in IL-10 in the 5–6 mo LPS-treated HIV-1Tg rats as well as the control and LPS-treated 18–20 mo old HIV-1Tg rats compared to the LPS-treated age-matched F344 rats (Fig. 5F). IL-1β in both the control HIV-1Tg and the F344 rats was below detection level for this assay (<3 pg/ml). The IL-1β levels in the LPS-treated F344 and HIV-1Tg rats declined with increased age (Fig. 5G). KC/GRO levels were low in both control HIV-1Tg and F344 rats; however, KC/GRO levels increased significantly in 5–6 and 18–20 mo old LPS-treated HIV-1Tg rats compared to age-matched LPS-treated F344 rats (Fig. 5H). Both IL-6 and TNF-α were minimal at all ages in the control HIV-1Tg and F344 animals. There was a significant increase in both the IL-6 and TNF-α levels in the serum of LPS-treated HIV-1Tg rats at 18–20 mo of age compared to the age-matched control and LPS-treated F344 rats (Figs. 5I and 5J).

**Figure 6:**
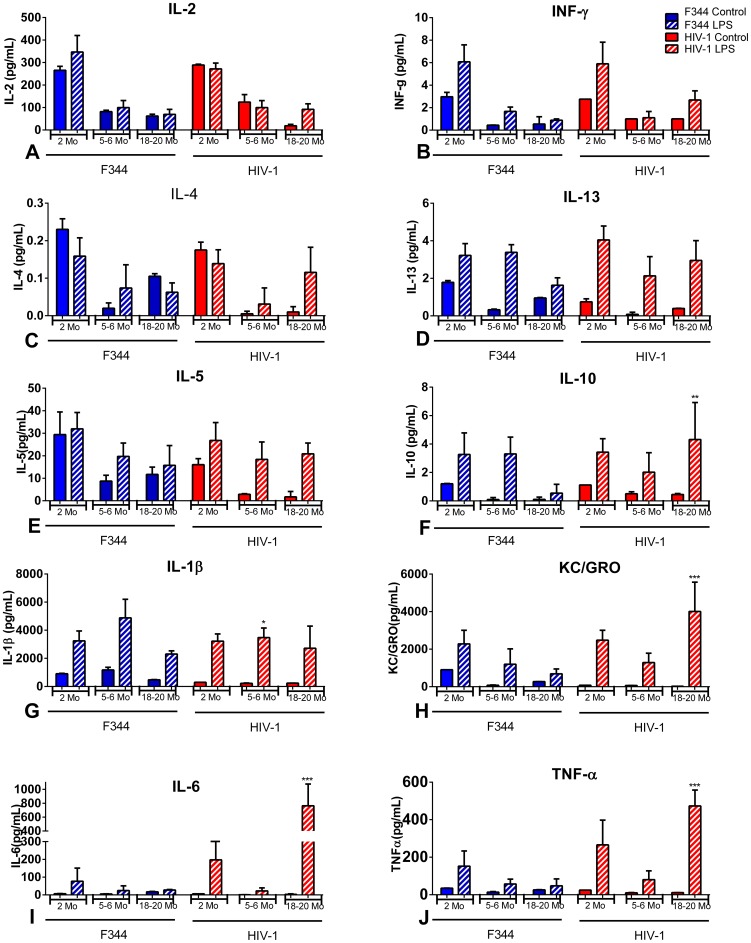
Cytokines and chemokines in the spleens of aging HIV-1Tg and F344 rats, with and without LPS treatment. Cytokine and chemokine levels in the spleen of HIV-1Tg rats (red bars) and F344 age-matched control rats (blue bars). Solid bars indicate control samples and bars with striped lines indicate samples treated with LPS. (A) IL-2; (B) IFN-γ; (C) IL-4; (D) IL-13; (E) IL-5; (F) IL-10; (G) IL-1β; (H) KC/GRO; (I) IL-6; (J) TNF-α. *p<0.05, **p<0.01, ***p<0.001, ****p<0.0001.

Spleen samples showed similar levels of IL-2, INF-γ, IL-4, and IL-5 in age- and treatment-matched HIV-1Tg and F344 rats (Figs. 6A, B, C, and E). IL-13 increased in response to LPS in both the F344 and HIV-1Tg rats (Fig. 6D). The increase in IL-10 in response to LPS was similar in the 2 and 5–6 mo old F344 rats, but had declined at 18–20 mo of age (Fig. 6F). IL-10 in the HIV-1Tg rats was increased in response to LPS at all ages, and was significantly higher at 18–20 mo of age compared to age-matched F344 animals (Fig. 6F). IL-1β levels were comparable in the spleen of control HIV-1Tg and F344 rats, and increased in response to LPS in both groups. However, at 5–6 mo of age, the level of IL-1β was significantly lower in the LPS-treated HIV-1Tg rats compared to the LPS-treated age-matched F344 rats (Fig. 6G). KC/GRO levels were similar in control HIV-1Tg and F344 rats, and increased in response to LPS in both groups. The LPS-induced levels of KC/GRO decreased with age in the F344 rats, whereas KC/GRO remained significantly higher in the 18–20 mo old HIV-1Tg rats (Fig. 6H). IL-6 and TNF-α were at basal levels in the spleens of the control HIV-1Tg and F344 rats. Both IL-6 and TNF-α increased significantly in response to LPS in the spleens of the 18–20 mo old HIV-1Tg rats compared to the age- and treatment-matched F344 rats (Figs. 6I and 6J).

**Figure 7:**
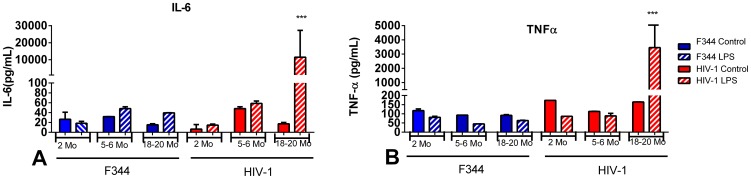
IL-6 and TNF-α in lymph nodes of aging HIV-1Tg and F344 rats, with and without LPS treatment. Pro-inflammatory cytokines, (A) IL-6 and (B) TNF-α, were examined in the lymph nodes of HIV-1Tg (red bars) and age-matched F344 control rats (blue bars), with and without LPS treatment. Solid bars indicate control samples and bars with striped lines indicate samples treated with LPS. *p<0.05, **p<0.01, ***p<0.001, ****p<0.0001.

Low levels of IL-6 and TNF-α were found in the lymph nodes in control HIV-1Tg and F344 rats as well as in 2 and 5–6 mo old LPS-treated HIV-1Tg and F344 animals (Figs. 7A and B). There were significantly higher levels of IL-6 and TNF-α in the lymph nodes of LPS-treated 18–20 mo old HIV-1Tg rats compared to age-matched LPS-treated F344 rats.

### IL-6 and TNF-α protein analysis

**Figure 8:**
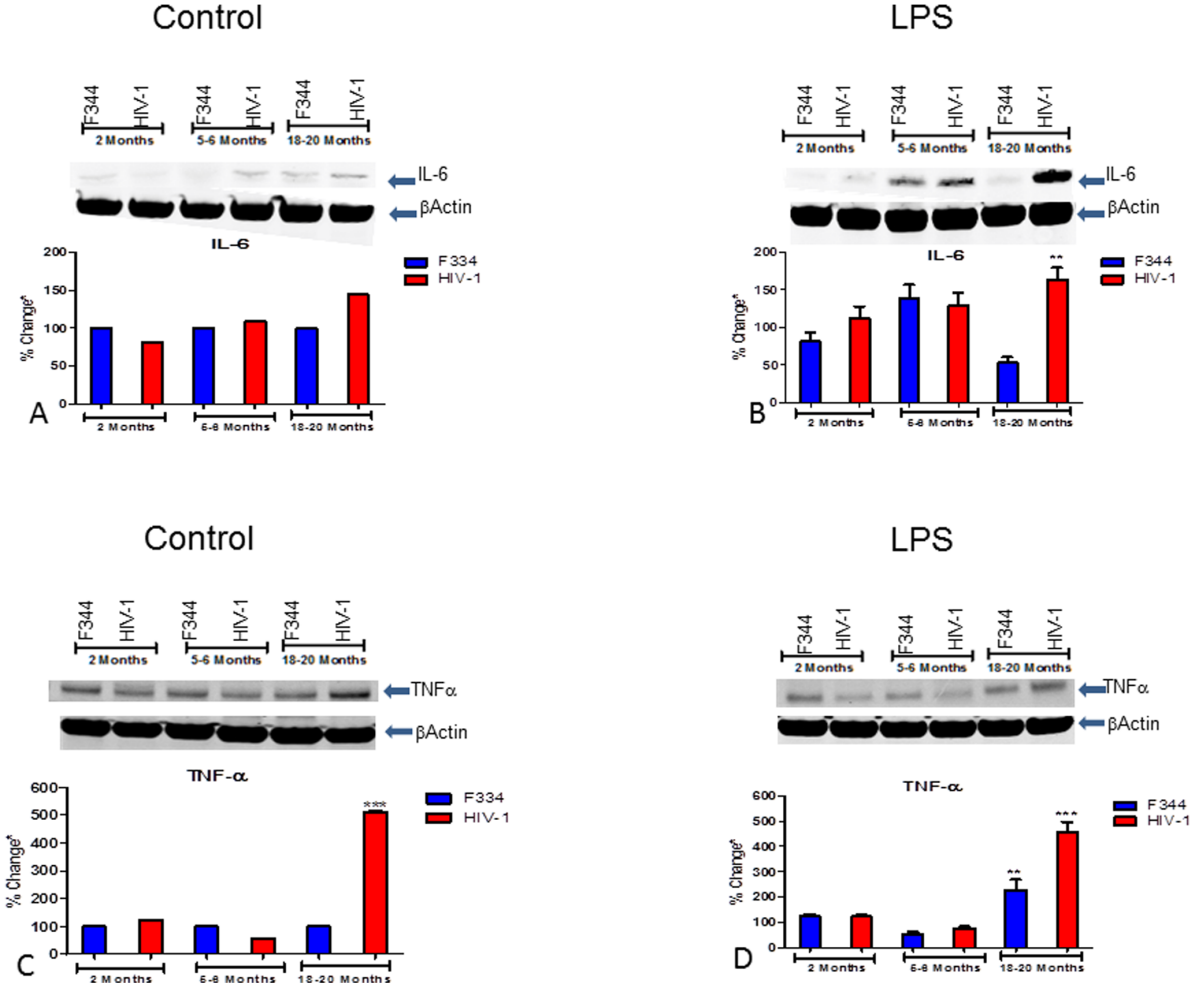
IL-6 and TNF-α in the spleens of aging HIV-1Tg and F344 rat spleen, with and without LPS treatment. The integrated intensity of TNF-α and IL-6 protein from the spleens of HIV-1Tg (red bars) and F344 age-matched control rats (blue bars), with and without LPS treatment, was measured using Western blot analysis and normalized to β-actin protein. (A) IL-6 after saline treatment; (B) IL-6 after LPS treatment; (C) TNF-α after saline treatment; (D) TNF-α after LPS treatment. *p<0.05, **p<0.01, ***p<0.001, ****p<0.0001.

The IL-6 protein signals were comparable in the control 2 and 5–6 mo old HIV-1Tg and F344 rats; however, there was a slight increase in the IL-6 signal in the spleens of the control 18–20 mo old HIV-1Tg rats compared to the-age matched F344 rats (Fig. 8A). IL-6 protein increased with age in the LPS-treated HIV-1Tg rats, with a significant increase seen in the 18–20 mo old HIV-1Tg rats compared to the age-matched LPS-treated F344 rats (Fig. 8B).

There was no significant difference in TNF-α levels in the spleens of the 2 and 5–6 mo control HIV-1Tg rats, but a significant increase in TNF-α levels in the spleens of the 18–20 mo old HIV-1Tg control rats (Fig. 8C); there was no significant difference in the 2, 5–6, and 18–20 mo control F344 animals. There was no difference in the TNF-α protein levels following LPS treatment in the 2 mo old HIV-1Tg and F344 rats, but a slight decrease in TNF-α protein levels in both groups at 5–6 mo of age. There was a significant increase in TNF-α in both the LPS-treated HIV-1Tg and F344 rats at 18–20 mo of age compared to the age-matched control F344 rats (Fig. 8D).

## Discussion

Aging itself is an inflammatory process. Aging causes alterations in immune responses, in general, with increased susceptibility to many infectious diseases [29]–[34]. Delineating the changes in immune responses in HIV-positive individuals is further complicated when factors such as age are taken into consideration. Utilizing the HIV-1Tg rat, we were able to demonstrate that, in this animal model of human HIV-1-positive patients receiving HAART, alterations in immune cell populations and in response to a LPS challenge occur with aging.

Flow cytometric analysis of untreated blood revealed that, in the HIV-1Tg rat, there is a decrease in T cells, particularly T cytotoxic cells, but an increase in T helper cells with increased age. Interestingly, the untreated F344 rats did not display changes in the percentage of T cells with aging, maintaining percentages of 52–55%. This result is similar to a study by Schmucker *et al*. (16) in which they compared young adult (4–6 mo old) and senescent (24–26 mo old) F344 rats and found consistent percentages of T cells in the blood regardless of age. Our data suggest that age may play a role in the decrease in T cells in the HIV-1Tg rats that is not seen in the F344 animals.

In healthy humans, CD14^+^CD16^−^ cells represent 90–95% of the total monocyte population in the blood. These cells are described as “classical”, whereas the remaining 5–10% are pro-inflammatory monocytes (CD14^+^CD16^+^) termed “non-classical” [35]. In the rat, two subtypes of monocytes have been described based on chemokine receptor expression and the presence and amount of CD43. CD43^+^ cells are similar to human CD14^+^CD16^−^ classical monocytes, whereas, in the rat, CD43^++^ monocytes act like human non-classical CD14^+^CD16^+^ populations [35], [36]. Monocytes are precursors for macrophage and dendritic cell populations and have important functions in innate and adaptive immunity [37]. Increases in non-classical monocytes have been associated with aging and with infections such as HIV. Non-classical monocytes produce higher amounts of inflammatory cytokines, including TNF-α, whereas classical monocytes produce a broad range of cytokines, including IL-10 and IL-6 [38]. The percentages of monocytes in this study were decreased in the untreated HIV-1Tg rats compared to the F344 animals, but increased with age. Interestingly, the percentage of classical and non-classical monocytes was similar throughout aging in both the HIV-1Tg and F344 rats. It is possible that the changes in the monocyte population and in cytokine production in the aging HIV-1Tg rats may be related to alterations in the macrophage or dendritic populations, which were not assayed in this study.

Our results demonstrated that untreated HIV-1Tg rats display alterations in immunophenotype upon aging that are not evident in age-matched F344 rats. Thus, the persistent presence of HIV-1 viral proteins appears to be involved in age-dependent immunomodulation.

LPS is a glycolipid component of gram-negative bacteria commonly used to study inflammatory responses as well as cytokine and chemokine changes [39]–[41]. A balance in pro-inflammatory and anti-inflammatory responses is imperative in achieving an appropriate immune response to a stimulus. An imbalance between pro- and anti-inflammatory cytokines can cause adverse biological events, such as septic shock [41]. Repeated exposure to an endotoxin such as LPS can cause endotoxin tolerance (ET), a state in which there is an increase in anti-inflammatory cytokines and a decrease in pro-inflammatory cytokines [41]. A recent study reported that ET causes an imbalance in the cytokine/chemokine response in an HIV-1Tg animal [27]. While innate immunity is deregulated in both HIV infection and ET [42], identifying changes in immune responses that are also related to age may provide useful information in the treatment of bacterial and other infections in the HIV patient.

Aging is linked with heightened amounts of circulating cytokines and pro-inflammatory markers [43]. Increased levels of the pro-inflammatory cytokines, IL-6 and TNF-α, along with IL-1 and C-reactive protein, have been associated with an elevated risk of morbidity and mortality found in the aging population [43]. In humans, a condition referred to as ‘inflamm-aging’, is associated with the increase in IL-6 and TNF-α that occurs with aging [44], [45]. In mature HIV-positive individuals, elevated levels of IL-6 and TNF-α have been reported [46]. Immunoscenescence, or aging of the immune system, caused by a prolonged antigen burden could be accelerated in HIV-positive individuals [47]–[49].

IL-6 is secreted by T cells and macrophages and has a role in inflammation and aging, whereas TNF-α is mainly secreted by macrophages [43]. KC/GRO is produced by macrophages and is involved in neutrophil activation. In our study, there was a significant increase in the percentage of neutrophils in the blood of untreated 18 mo HIV-1Tg rats and an increase in monocyte populations with aging. IL-10 inhibits the synthesis of many cytokines that have a role in suppression of Th1 pro-inflammatory responses and phagocytic uptake. The cytokines, IL-6 and TNF-α, along with the CXC chemokine, KC/GRO, were significantly elevated in the blood and spleen of 18–20 mo old LPS-treated HIV-1Tg rats. In addition, there was increased IL-6 and TNF-α protein expression in the lymph nodes. IL-10 levels were increased in the spleen of LPS-treated 18–20 mo old HIV-1Tg rats compared to LPS-treated age-matched F344 rats. Together, these results further indicate that age may play a role in the alterations in immune cell responses in HIV-positive individuals.

In HIV-infected patients, anti-retroviral therapies have been successful in controlling viral replication, allowing those individuals to achieve a relatively normal life span [50]–[52]. Viral proteins, however, are capable of independently inducing organ dysfunction and affecting host target cells (T cells and macrophages), resulting in the clinical manifestations of AIDS [19], [21], [53]. As an HIV-infected individual matures, immune cell function may be compromised even more. Age may, therefore, be a factor in determining appropriate therapies for HIV-infected patients.

Our results showed that the HIV-1Tg rat exhibits alterations in the percentages of T cells, neutrophils, and monocytes with aging. Thus, in addition to serving as a rodent neuroAIDS model in which to study HIV patients given HAART, this study suggests that the HIV-1Tg rat could also be an ideal model in which to study aging-associated immune alterations in HIV patients.

## Conclusion

The findings from this study provide evidence pointing to age-related alterations in immune cell function in the HIV-positive population. This information could be important in the development of novel therapeutic strategies for treating HIV-infected individuals based on multiple factors, including immune cell profile, cellular responses, and age of the patient.

## References

[1] High KP, Brennan-Ing M, Clifford DB, Cohen MH, Currier J, et al (2012) HIV and aging: state of knowledge and areas of critical need for research. A report to the NIH Office of AIDS Research by the HIV and Aging Working Group. J Acquir Immune Defic Syndr 60 Suppl 1 S1–18. 22688010 10.1097/QAI.0b013e31825a3668 PMC3413877

[2] Mills EJ, Barnighausen T, Negin J (2012) HIV and aging–preparing for the challenges ahead. N Engl J Med 366: 1270–1273. 22475591 10.1056/NEJMp1113643

[3] Summary report from the Human Immunodeficiency Virus and Aging Consensus Project: treatment strategies for clinicians managing older individuals with the human immunodeficiency virus. J Am Geriatr Soc 60: 974–979. 22568508 10.1111/j.1532-5415.2012.03948.x

[4] Nath A (2012) HIV and aging. J Neurovirol 18: 245–246. 22623169 10.1007/s13365-012-0111-4 PMC4902660

[5] (2012) Health Protection Agency. HIV in the United Kingdom. 2012 Report. London: Colindale.

[6] Lucas GM, Mehta SH, Atta MG, Kirk GD, Galai N, et al (2007) End-stage renal disease and chronic kidney disease in a cohort of African-American HIV-infected and at-risk HIV-seronegative participants followed between 1988 and 2004. Aids 21: 2435–2443. 18025880 10.1097/QAD.0b013e32827038ad

[7] Silverberg MJ, Chao C, Leyden WA, Xu L, Tang B, et al (2009) HIV infection and the risk of cancers with and without a known infectious cause. Aids 23: 2337–2345. 19741479 10.1097/QAD.0b013e3283319184 PMC2863991

[8] Valcour V, Sithinamsuwan P, Letendre S, Ances B (2011) Pathogenesis of HIV in the central nervous system. Curr HIV/AIDS Rep 8: 54–61. 21191673 10.1007/s11904-010-0070-4 PMC3035797

[9] Nath A, Schiess N, Venkatesan A, Rumbaugh J, Sacktor N, et al (2008) Evolution of HIV dementia with HIV infection. Int Rev Psychiatry 20: 25–31. 18240060 10.1080/09540260701861930

[10] Freiberg MS, Chang CC, Skanderson M, McGinnis K, Kuller LH, et al (2011) The risk of incident coronary heart disease among veterans with and without HIV and hepatitis C. Circ Cardiovasc Qual Outcomes 4: 425–432. 21712519 10.1161/CIRCOUTCOMES.110.957415 PMC3159506

[11] Womack JA, Goulet JL, Gibert C, Brandt C, Chang CC, et al (2011) Increased risk of fragility fractures among HIV infected compared to uninfected male veterans. PLoS One 6: e17217. 21359191 10.1371/journal.pone.0017217 PMC3040233

[12] Sulkowski MS, Thomas DL (2003) Hepatitis C in the HIV-Infected Person. Ann Intern Med 138: 197–207. 12558359 10.7326/0003-4819-138-3-200302040-00012

[13] Ropolo A, Moron VG, Maletto B, Pistoresi-Palencia MC (2001) Diminished percentage of antigen bearing cells in the lymph nodes of immune aged rats. Exp Gerontol 36: 519–535. 11250123 10.1016/s0531-5565(00)00222-9

[14] Pahlavani MA, Vargas DA (2001) Aging but not dietary restriction alters the activation-induced apoptosis in rat T cells. FEBS Lett 491: 114–118. 11226431 10.1016/s0014-5793(01)02184-6

[15] Dawson HD, Ross AC (1999) Chronic marginal vitamin A status affects the distribution and function of T cells and natural T cells in aging Lewis rats. J Nutr 129: 1782–1790. 10498748 10.1093/jn/129.10.1782

[16] Schmucker DL, Owen TM, Issekutz TB, Gonzales L, Wang RK (2002) Expression of lymphocyte homing receptors alpha4beta7 and MAdCAM-l in young and old rats. Exp Gerontol 37: 1089–1095. 12213559 10.1016/s0531-5565(02)00065-7

[17] Reid W, Sadowska M, Denaro F, Rao S, Foulke J Jr, et al (2001) An HIV-1 transgenic rat that develops HIV-related pathology and immunologic dysfunction. Proc Natl Acad Sci U S A 98: 9271–9276. 11481487 10.1073/pnas.161290298 PMC55410

[18] Reid W, Abdelwahab S, Sadowska M, Huso D, Neal A, et al (2004) HIV-1 transgenic rats develop T cell abnormalities. Virology 321: 111–119. 15033570 10.1016/j.virol.2003.12.010

[19] Joshi PC, Raynor R, Fan X, Guidot DM (2008) HIV-1-transgene expression in rats decreases alveolar macrophage zinc levels and phagocytosis. Am J Respir Cell Mol Biol 39: 218–226. 18314538 10.1165/rcmb.2007-0344OC PMC2542456

[20] Chang SL, Beltran JA, Swarup S (2007) Expression of the mu opioid receptor in the human immunodeficiency virus type 1 transgenic rat model. J Virol 81: 8406–8411. 17553897 10.1128/JVI.00155-07 PMC1951376

[21] Peng J, Vigorito M, Liu X, Zhou D, Wu X, et al (2010) The HIV-1 transgenic rat as a model for HIV-1 infected individuals on HAART. J Neuroimmunol 218: 94–101. 19913921 10.1016/j.jneuroim.2009.09.014

[22] Homji NF, Vigorito M, Chang SL (2012) Morphine-induced conditioned place preference and associated behavioural plasticity in HIV-1 transgenic rats. Int J Clin Exp Med 5: 105–123. 22567172 PMC3342709

[23] Chang SL, Connaghan KP (2012) Behavioral and molecular evidence for a feedback interaction between morphine and HIV-1 viral proteins. J Neuroimmune Pharmacol 7: 332–340. 22083500 10.1007/s11481-011-9324-1

[24] Yadav A, Pati S, Nyugen A, Barabitskaja O, Mondal P, et al (2006) HIV-1 transgenic rat CD4+ T cells develop decreased CD28 responsiveness and suboptimal Lck tyrosine dephosphorylation following activation. Virology 353: 357–365. 16828835 10.1016/j.virol.2006.05.026

[25] Zhou X, Luo YC, Ji WJ, Zhang L, Dong Y, et al (2013) Modulation of mononuclear phagocyte inflammatory response by liposome-encapsulated voltage gated sodium channel inhibitor ameliorates myocardial ischemia/reperfusion injury in rats. PLoS One 8: e74390. 24069305 10.1371/journal.pone.0074390 PMC3777990

[26] Sengupta P (2013) The Laboratory Rat: Relating Its Age With Human's. Int J Prev Med 4: 624–630. 23930179 PMC3733029

[27] Homji NF, Mao X, Langsdorf EF, Chang SL (2012) Endotoxin-induced cytokine and chemokine expression in the HIV-1 transgenic rat. J Neuroinflammation 9: 3. 22216977 10.1186/1742-2094-9-3 PMC3322344

[28] Chen R, Zhou H, Beltran J, Malellari L, Chang SL (2005) Differential expression of cytokines in the brain and serum during endotoxin tolerance. J Neuroimmunol 163: 53–72. 15885308 10.1016/j.jneuroim.2005.02.012

[29] Aberg JA (2011) Aging, inflammation, and HIV infection. Top Antivir Med 20: 101–105. PMC6148943 22954610

[30] Barrett L, Fowke KR, Grant MD (2012) Cytomegalovirus, aging, and HIV: a perfect storm. AIDS Rev 14: 159–167. 22833059

[31] Cavanagh MM, Weyand CM, Goronzy JJ (2012) Chronic inflammation and aging: DNA damage tips the balance. Curr Opin Immunol 24: 488–493. 22565047 10.1016/j.coi.2012.04.003 PMC3423478

[32] Kogut I, Scholz JL, Cancro MP, Cambier JC (2012) B cell maintenance and function in aging. Semin Immunol 24: 342–349. 22560930 10.1016/j.smim.2012.04.004

[33] Rymkiewicz PD, Heng YX, Vasudev A, Larbi A (2012) The immune system in the aging human. Immunol Res 53: 235–250. 22477521 10.1007/s12026-012-8289-3

[34] Goronzy JJ, Weyand CM (2012) Immune aging and autoimmunity. Cell Mol Life Sci 69: 1615–1623. 22466672 10.1007/s00018-012-0970-0 PMC4277694

[35] Strauss-Ayali D, Conrad SM, Mosser DM (2007) Monocyte subpopulations and their differentiation patterns during infection. J Leukoc Biol 82: 244–252. 17475785 10.1189/jlb.0307191

[36] Grau V, Scriba A, Stehling O, Steiniger B (2000) Monocytes in the rat. Immunobiology 202: 94–103. 10879693 10.1016/S0171-2985(00)80056-X

[37] Tacke F, Randolph GJ (2006) Migratory fate and differentiation of blood monocyte subsets. Immunobiology 211: 609–618. 16920499 10.1016/j.imbio.2006.05.025

[38] Wong KL, Tai JJ, Wong WC, Han H, Sem X, et al (2011) Gene expression profiling reveals the defining features of the classical, intermediate, and nonclassical human monocyte subsets. Blood 118: e16–31. 21653326 10.1182/blood-2010-12-326355

[39] Schletter J, Heine H, Ulmer AJ, Rietschel ET (1995) Molecular mechanisms of endotoxin activity. Arch Microbiol 164: 383–389. 8588739 10.1007/BF02529735

[40] Fujihara M, Muroi M, Tanamoto K, Suzuki T, Azuma H, et al (2003) Molecular mechanisms of macrophage activation and deactivation by lipopolysaccharide: roles of the receptor complex. Pharmacol Ther 100: 171–194. 14609719 10.1016/j.pharmthera.2003.08.003

[41] Biswas SK, Lopez-Collazo E (2009) Endotoxin tolerance: new mechanisms, molecules and clinical significance. Trends Immunol 30: 475–487. 19781994 10.1016/j.it.2009.07.009

[42] Lester RT, Yao XD, Ball TB, McKinnon LR, Omange WR, et al (2009) HIV-1 RNA dysregulates the natural TLR response to subclinical endotoxemia in Kenyan female sex-workers. PLoS One 4: e5644. 19461969 10.1371/journal.pone.0005644 PMC2680984

[43] Michaud M, Balardy L, Moulis G, Gaudin C, Peyrot C, et al (2013) Proinflammatory cytokines, aging, and age-related diseases. J Am Med Dir Assoc 14: 877–882. 23792036 10.1016/j.jamda.2013.05.009

[44] Franceschi C, Bonafe M, Valensin S, Olivieri F, De Luca M, et al (2000) Inflamm-aging. An evolutionary perspective on immunosenescence. Ann N Y Acad Sci 908: 244–254. 10911963 10.1111/j.1749-6632.2000.tb06651.x

[45] Wolf J, Weinberger B, Arnold CR, Maier AB, Westendorp RG, et al (2012) The effect of chronological age on the inflammatory response of human fibroblasts. Exp Gerontol 47: 749–753. 22790019 10.1016/j.exger.2012.07.001 PMC3427851

[46] Nixon DE, Landay AL (2010) Biomarkers of immune dysfunction in HIV. Curr Opin HIV AIDS 5: 498–503. 20978393 10.1097/COH.0b013e32833ed6f4 PMC3032605

[47] Franceschi C, Capri M, Monti D, Giunta S, Olivieri F, et al (2007) Inflammaging and anti-inflammaging: a systemic perspective on aging and longevity emerged from studies in humans. Mech Ageing Dev 128: 92–105. 17116321 10.1016/j.mad.2006.11.016

[48] Vasto S, Candore G, Balistreri CR, Caruso M, Colonna-Romano G, et al (2007) Inflammatory networks in ageing, age-related diseases and longevity. Mech Ageing Dev 128: 83–91. 17118425 10.1016/j.mad.2006.11.015

[49] Smith RL, de Boer R, Brul S, Budovskaya Y, van Spek H (2013) Premature and accelerated aging: HIV or HAART? Front Genet 3: 328. 23372574 10.3389/fgene.2012.00328 PMC3556597

[50] Manfredi R (2004) Impact of HIV infection and antiretroviral therapy in the older patient. Expert Rev Anti Infect Ther 2: 821–824. 15566324 10.1586/14789072.2.6.821

[51] Gebo KA (2006) HIV and aging: implications for patient management. Drugs Aging 23: 897–913. 17109568 10.2165/00002512-200623110-00005

[52] Manfredi R, Calza L (2004) [HIV infection and AIDS in advanced age. Epidemiological and clinical issues, and therapeutic and management problems]. Infez Med 12: 152–173. 15711129

[53] van Maanen M, Sutton RE (2003) Rodent models for HIV-1 infection and disease. Curr HIV Res 1: 121–130. 15043216 10.2174/1570162033352075

